# The association between long-distance migration and PTSD prevalence in Syrian refugees

**DOI:** 10.1186/s12888-022-03982-4

**Published:** 2022-05-27

**Authors:** Andreas Halgreen Eiset, Michaelangelo P. Aoun, Monica Stougaard, Annemarie Graa Gottlieb, Ramzi S. Haddad, Morten Frydenberg, Wadih J. Naja

**Affiliations:** 1 Department of Affective Disorders, Aarhus University Hospital–Psychiatry, Palle Juul Jensens Boulevard 175, 8200 Aarhus N Aarhus, Denmark; 2 Department of Public Health, Aarhus University, Bartholins Allé 2, 8000 Aarhus, Denmark; 3 Faculty of Medical Sciences, Lebanese University, P.O. Box 6573/14, Badaro, Museum, Beirut, Lebanon; 4 Consultant Biostatistician, Høgemosevej 19A, Olsted, 8380 Trige, Denmark; 5 King Hussein Cancer Center, Queen Rania St 202, Amman, Jordan

**Keywords:** Post-traumatic stress disorder, PTSD, Refugees, Human migration, Emigration and immigration, Propensity score, Cross-sectional studies

## Abstract

**Background:**

Refugees are forced migrants but there is a large variation in the distance that refugees cover and there is a paucity in the evidence of how this may affect refugees’ health and health care needs. Objective: We investigated the association between long-distance migration and post-traumatic stress disorder (PTSD), a serious psychiatric disorder associated with deteriorating mental and somatic health.

**Methods:**

Included from 2016–2019 were adult Syrian refugees in Lebanon and Denmark that arrived up to 12 months prior to inclusion. PTSD was assessed using the Harvard Trauma Questionnaire and the estimate of association was obtained by multiply imputing missing data and adjusting for confounding by propensity score-weighting with covariates age, sex, socioeconomic status, trauma experience and general mental well-being, reporting the bootstrap 95-percentile confidence interval (95% CI). Additionally, a number of sensitivity analyses were performed.

**Results:**

Included were 599 participants in Lebanon (mean age 35 years old, 73% being female) and 133 participants in Denmark (mean age 30 years old, 47% being female). After multiply imputing missing data and propensity score-weighted adjustment for confounding, migration to Denmark instead of Lebanon was associated with an increase in PTSD prevalence of 9 percentage point (95% CI [-1; 19] percentage point).

**Conclusions:**

Long-distance migration may be associated with an increase in PTSD prevalence in refugees. The migration could be an important factor to consider when assessing refugees’ and asylum seekers’ health. Practitioners should consider “long-distance migration” in refugee health screenings and in particular when assessing the risk of post-traumatic stress disorder. Future research should be designed to ultimately lead to studies of relevant interventions to lower the risk of post-traumatic stress disorder in refugees.

**Supplementary Information:**

The online version contains supplementary material available at 10.1186/s12888-022-03982-4.

## Background

Post-traumatic stress disorder (PTSD) is a severe psychiatric disorder that is associated with significant loss of functioning and increased morbidity and mortality [1–3]. Risk factors include multiple traumas, female sex, being spouseless, lower educational status, comorbid disease and type of trauma [3, 4]. Also, gene-environment interactions may play a key role [5]. Refugees may be exposed to risk-factors for PTSD before, during and after migration (e.g. trauma, acquiring comorbid disease, low socioeconomic status, psychiatric co-morbidity) [6–8]; however, compared with the general population in the country of origin refugees in western countries on average have lower burden of several risk factors for PTSD such as higher educational status and lower comorbidity [8–11]. There is little empirical evidence on pre- and peri-migratory morbidity in refugees probably due to difficulty in data acquisition. There is, however, an abundance of publications on post-migratory morbidity in refugees, for example, the prevalence of latent tuberculosis is reported between 15 and 45% [12] and the prevalence of depression between 3 and 58% in a recent meta-analysis [13]. The post-migratory prevalence of PTSD across refugee populations was estimated to 31% [13] and about three times higher than in the general population in the host country [3, 14] although, with considerable variation between country of origin (e.g. in Australia, the prevalence was 21% in refugees from Africa and Asia and 63% in refugees from former Yugoslavia) and host country (e.g. in refugees from Iraq, the prevalence was 43% in Turkey and 4% in USA). To design appropriate interventions to counter this disparity in health, the drivers of the disparity must be understood [15, 16]; however, there is a paucity of evidence on one of the most fundamental factors shared by refugees: the migration itself. In particular, there is a knowledge gap in the health effects of the distance between the country of origin and the host country. The concept of “distance” and the association between long-distance migration and a number of non-health outcomes has received some attention [17–20]. Recently, Spolaore and Warcziarg [21] used data from the World Values Survey to produce estimates of the distance between pairs of countries and showed that this was correlated with amongst others genetic distance (a measure of relatedness between populations based on gene frequency). Detollenaere et al*.* [22] used these distance estimates to show that long-distance migration was associated with lower self-rated health in migrant populations with mean length of stay > 20 years in European host countries. Specifically for the association between PTSD and long-distance migration recent studies of Syrian refugees in northern European and middle eastern countries have been ambiguous [23, 24], however, were hampered by confounding and non-randomised sample designs. Here, we present a cluster-randomised potential outcomes study in newly arrived Syrian refugees in Lebanon and newly arrived Syrian asylum seekers in Denmark to estimate the association between long-distance migration and PTSD prevalence minimising by design the effect of living in the host country. We hypothesised an increased PTSD prevalence after long-distance migration.

## Methods

In a cross-sectional design with one-stage cluster randomised sampling between 2016 through 2019, newly arrived Syrian refugees were included in Lebanon and newly arrived Syrian asylum seekers were included in Denmark and assessed for PTSD. The implemented inclusion criteria were: (a) adult (≥ 18 years of age), (b) Syrian-born, (c) had left Syria after the onset of the ongoing civil-war (after February 2011), (d) arrived in host country less than 12 months prior to inclusion and (e) resident at the inclusion site at the time of inclusion. Exclusion criteria were physical or mental illness that prevented participation. The study was performed in accordance with the Declaration of Helsinki and was approved by all appropriate ethics committees. We report in accordance with the STROBE guideline for reporting observational studies [25] (Supplementary Material 1).

Lebanon is a country of about 6 million people living in an area of nearly 10.000 km^2^, bordering Syria, the Mediterranean Sea and Israel. Since 2015 Lebanon has stopped accepting asylum applications from Syrians seeking refuge in Lebanon. Nevertheless, being a close neighbour, in both geographical, linguistic and cultural distance, the influx of Syrian refugees has endured with the majority living in structures ranging from regular (though dilapidated) structures to improvised tents clustered in informal gatherings and formal refugee camps scattered across Lebanon [26]. Denmark is a Northern European country of about 6 million people living in an area of about 50.000 km^2^. With few exceptions, an asylum seeker is allocated by random to one of the Danish asylum seeker centres. These centres are run by the Danish Immigration Services but outsourced to different operators. Several types of asylum seeker centres exist with the most common being the accommodation centre.

### Variables and data-collection instruments

The exposure was long-distance migration as indicated by the Spolaore and Warcziarg distance estimates [21]: migration from Syria to Lebanon was short-distance, i.e. “unexposed”, and migration from Syria to Denmark was long-distance, i.e. “exposed”. The outcome, PTSD, was assessed using the Harvard Trauma Questionnaire (HTQ): A self-administered scale to assess symptoms of PTSD in the past week according to the DSM-IV criteria [27] and validated in multiple settings, languages and populations [28–30]. Two recent studies that investigated the construct validity, factor structure and measurement invariance found that the HTQ is a reliable instrument in non-western populations in general [31] and Arabic speaking refugees in particular [32]. The DSM-IV score is the mean of 16 items scored from 1 (“not at all”) to 4 (“extremely”) and a cut-off ≥ 2.5 is often taken to indicate PTSD; however, it is debated whether this cut-off score is transportable across cultural settings [33]. The validated Arabic 2007 version [34] was used. The internal consistency for the HTQ was estimated by Cronbach’s alpha to 0.93.

The variables necessary to address confounding were decided upon a priori after discussions among the authors guided by graphical representation of our assumptions (Supplementary Fig. 1) and included age, sex, exposure to violence during migration (victim or witness), socioeconomic status in the country of origin, and general mental well-being. For the latter, the WHO-5 scale was used: a self-administered scale to assess “subjective positive well-being” [35, 36], validated across settings and cultures [36–38]. The five items are scored from 0 (“at no time”) to 5 (“all of the time”), summed and multiplied by 4; a cut-off score ≤ 50 indicates a risk of poor well-being, while scores < 13, or any one item-score of 0 or 1, indicates poor well-being and formal diagnostics for depression and mental health issues are recommended. The validated Arabic 1999-version [39] was used. All other variables were collected using a questionnaire (Supplementary Text 1) developed in Danish, translated by a bilingual and bicultural translator into Arabic (mother tongue Syrian Arabic) and back translated by two independent translators (mother tongue Arabic (other Arabic dialects than Syrian). Each item was evaluated and minor corrections were implemented. The final translated questionnaire was approved by MPA, RSH and WJN.

### Sample size, randomisation and data collection

Based on power calculations the aimed sample size was 1100 participants in Lebanon and 220 in Denmark [40]. In both Lebanon and Denmark cluster sampling was used and data was collected by teams of health care professionals (at least author AHE or MPA at any time) and students after several training sessions in practicalities and ethical considerations. In collaboration with local non-governmental organisations, Lebanon was stratified into five regions based on geography, infrastructure and insights about local concerns (political, religious etc.) and the sampling frame was drawn to include all formal and informal refugee camps, urban and rural communities, settlements and gatherings of Syrian refugees. Inclusion sites were chosen at random from within each strata (i.e. stratified cluster sampling) and all individuals eligible for inclusion, as determined by oral communication, were invited to participate securing proportional allocation. Permission to include participants in all regions of Lebanon was obtained, including areas that are usually outside the reach of researchers due to “security considerations”. In case of unsafe conditions at the scheduled area and time of inclusion the closest setting deemed appropriate within the same region was used. In Denmark, all five operators of accommodation centres were invited to participate. Three responded to the invitation, all accepting, representing a total of 11 accommodation centres. All eligible residents at six randomly sampled accomodation centres were invited. After informed oral and written consent, participants were instructed to independently complete the background questionnaire and the mental health scales. The data collectors assisted those who could not read or write or had further questions regarding the study. Participants could at any time opt out of the data collection. Individuals that refused to participate were asked for a short non-structured interview including basic demographics and reason for refusal. All participants that needed immediate medical or psychiatric assistance were referred to the local operators in Lebanon or Denmark. The procedure was piloted in Denmark. All relevant permissions were obtained prior to inclusion of any participant in both Lebanon and Denmark. The sampling frame, sample size estimation for each region and training of the team of data collectors, is detailed in the study protocol [40].

### Statistical analysis

The analysis plan was specified a priori and is supplied at https://github.com/eiset/ARCH together with a number of exploratory plots and the R code. In the following we give a brief summary of the applied statistical methodology and refer to Supplementary Text 2, Supplementary Tables 1–2 and Supplementary Figs. 3, 4, 5 for further details as well as [41] for an in-depth discussion of its implications and usage. Missing data was multiply imputed after verifying for each partly observed variable that the data was “everywhere missing at random” (see Supplementary Text 2 and [42]. For each partly observed variable missing values were imputed using the substantive model and additional auxiliary variables that were known or empirically shown to be predictors of the given variable. This resulted in a “response-and-predictor matrix” as presented in Supplementary Table 2. Confounding was addressed by propensity score-weighting: The “standardised mortality ratio”-weights [43] was computed to estimate the weighted prevalence difference of PTSD in the population that migrated to Denmark instead of Lebanon and presented with a 95-percentile confidence interval found by bootstrapping. Three exposure models of increasing complexity were proposed (Supplementary Table 1) and three levels of truncation of extreme weights implemented (none, at 1st and 99th percentile and at 5th and 95th percentile). The simplest model with least truncation where all covariates had an absolute standardized mean difference below 10% was to be used as the model for the main analysis. To evaluate the robustness of the results of the analysis, the following sensitivity analyses were planned:

- Evaluating the model choice for the propensity score analysis using other propensity score models (Supplementary Table 1) that obtained acceptable balance on all covariates.
- The cut-off value of the HTQ score to indicate PTSD was set to the “standard” ≥ 2.5. The estimates and corresponding 95% CI from models with cut-off values at ≥ 2.3 and ≥ 2.7 is supplied.
- Assessing the impact of non-ignorable missingness mechanism by forcing all missing in the “socio-economic status” covariate in participants from Lebanon to be “Do not know/refuse to answer” and forcing all missing in the “Experienced violence during migration” covariate in participants from Denmark to “Yes”. In both scenarios all other missing were kept as imputed.

In addition “consonance plots” [44] were produced to allow for visualisation of the compatibility of hypothesised point estimates with the data. All data management, analysis and plots were done in R [45] with heavy reliance on the “Tidyverse” packages [46] for data management and plots, “smcfcs” [47] for multiple imputation, “WeightIt” [48] for obtaining the propensity score weights, “boot” [49] for parallelised bootstrapping, and “furrr” [50] for further parallelising.

## Results

**Fig. 1:**
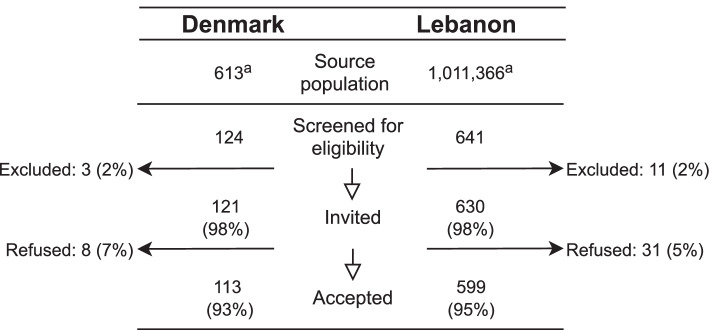
Flow-chart of the inclusion in Denmark and Lebanon. a The number given for the source population represents a maximum and include for example children. It was not possible to obtain a more precise estimate of the study population. b Refusals: predominantly males, predominantly age between 20 and 40, no difference between Lebanon and Denmark; however, sex was only recorded for 8 non-participants in Lebanon and few non-participants provided age (two in Denmark and two in Lebanon), thus a best guess is provided. Reasons for refusal: in Denmark 63% due to mistrust; in Lebanon predominantly no time, however, 77% did not answer

A total of 599 individuals were included in Lebanon (response rate 95%) and 113 were included in Denmark (response rate 93%). Figure 1 gives a statistical summary of the inclusion process and non-participation. In Lebanon, the mean duration of migration from Syria was one day, 60% had travelled by car and 30% had travelled by bus. In Denmark, the mean duration of migration was 44 days, 70% had travelled by boat, 60% had walked long distances and 50% had travelled by train. The mean length of stay in the host country at time of inclusion was 4 months in Lebanon and 6 months in Denmark.

**Fig. 2:**
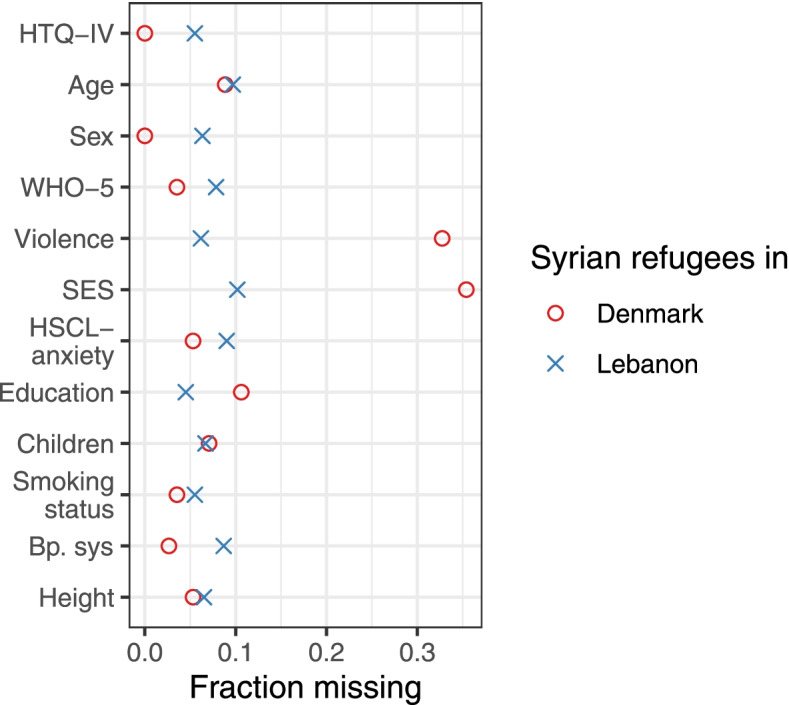
Missing fraction plot. Variables included in the propensity score and multiple imputation models for estimating the association between long-distance migration and PTSD among Syrian refugees in Lebanon and Denmark. Abbreviations: *HTQ-IV* Harvard Trauma Questionnaire part IV, *WHO-5* World Health Organization-5 Mental-health scale, Violence, exposure (directly or indirectly) to violence; *HSCL-anxeity* Hopkins Symptom Check List, anxiety part, *Bp.sys* systolic blood pressure

The study populations differed on key variables such as sex (female: 73% in Lebanon, 47% in Denmark) and experience of violence (24% in Lebanon, 39% in Denmark) and there were missing values for all variables except long-distance migration. Figure 2 illustrates the missing proportion of key variables stratified on exposure status. The assumptions for doing multiple imputation were met (see Supplementary Text 2) and Table 1 gives a detailed summary of the study population characteristics for both the observed realisation and the imputed data set including missing for each covariates in the observed data. Eight individuals (25% female) refused participating in Denmark while 31 individuals (16% female) refused participating in Lebanon, however few agreed to provide any information. In Denmark, the primary reason was mistrust and in Lebanon primarily reason was time concerns. Further details are given in Supplementary Table 3.

**Table 1:** Comparison of summary statistics of key variables in observed (with missing) and imputed data set stratified on exposure group

	**Observed data**		**Imputed data**	
	**Lebanon (*n* = 599)**	**Denmark (*n* = 113)**	**Lebanon (*n* = 599)**	**Denmark (*n* = 113)**
**Age—years old**	35 (20)	30 (12.5)	35 (20)	30 (12.9)
Missing	*n* = 58 (9.7%)	*n* = 10 (8.9%)	NA	NA
**Sex**				
Female	72.7%	46.9%	72.7%	46.9%
Missing	*n* = 38 (6.3%)	*n* = 0	NA	NA
**WHO-5-score**	24 (32)	24 (48)	24 (32)	24 (48)
Missing	*n* = 47 (7.9%)	*n* = 4 (3.5%)	NA	NA
**Experienced violence**				
Yes	23.8%	39.5%	23.8%	39.7%
Missing	*n* = 37 (6.2%)	*n* = 37 (32.7%)	NA	NA
**Socioeconomic status**				
Below average	25.5%	24.7%	25.3%	23.2%
On average	63.4%	50.7%	63.4%	50.0%
Above average^a^	4.5%	2.7%	11.3%^a^	26.8%^a^
Don’t know/refuse to answer^a^	6.7%	21.9%		
Missing	*n* = 61 (10.2%)	*n* = 40 (35.4%)	NA	NA
**HTQ score**	2.56 (0.94)	2.62 (1)	2.56 (0.95)	2.62 (1)
Missing	*n* = 33 (5.5%)	*n* = 0	NA	NA
**PTSD (HTQ score ≥ 2.5)**				
Overall	55.1%	60.2%	54.9%	60.2%
Female	59.4%	66.0%	59.1%	66.0%
Male	44.7%	55.0%	43.7%	55.0%
Missing	*n* = 33 (5.5%)	*n* = 0	NA	NA

Categorical variables are represented as % of observations. Continuous variables are represented as median (interquartile range). Missing is given as n (%) of all included participants for the observed data set

^a^In the substantive model, and thus in the multiple imputation, the strata “Above average” and “Don’t know/refuse to answer” was collapsed due to numerical problems in the multiple imputation

**Fig. 3:**
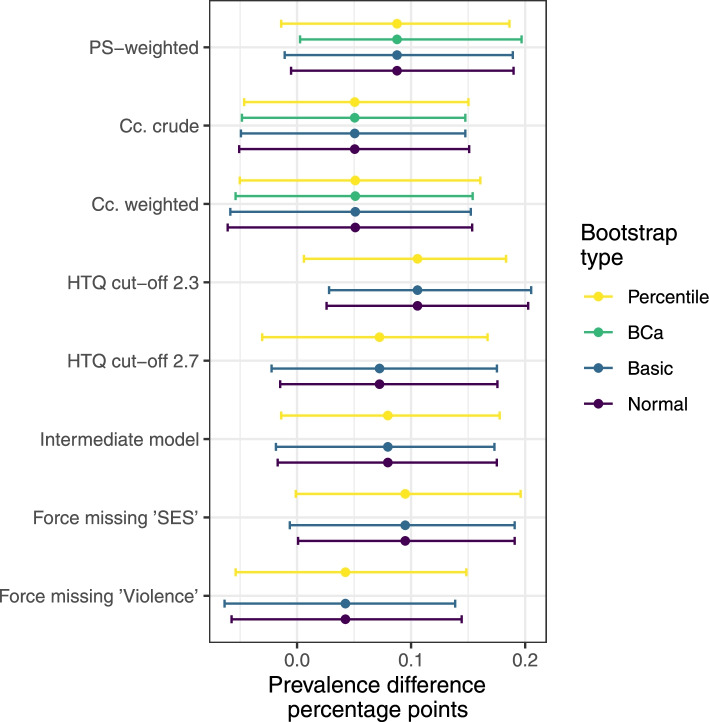
Estimates with different bootstrap confidence interval types. Abbreviations: *BCa* bias-corrected and accelerated bootstrap, *CI* CI confidence interval, *PS* propensity score, *Cc* complete case, *HTQ* Harvard Trauma Questionnaire part IV, *SES* socioeconomic status, Violence, exposure (directly or indirectly) to violence. From the top: the propensity score-weighted (“substantive model”) and the two complete case (no imputation) estimates with 999 bootstrap replications. The five sensitivity analysis are at the bottom, each with 250 bootstrap replications (and thus no BCa 95% CI)

The unadjusted prevalence of PTSD was higher in Denmark (60.2%) compared with Lebanon (55.1%). The prevalence difference of PTSD increased from 5.1 percentage point (95-percentile CI [-4.6; 15.0]) to 8.8 percentage point (95-percentile CI [-1.4; 18.6 percentage point]) after multiply imputing missing data and adjusting for confounding by propensity score-weighting. All sensitivity analyses produced estimates in the same direction and of the same magnitude, except when forcing all missing in the “Violence” variable to “Yes” in the study population included in Denmark, thus, grossly violating the missing-at-random assumption of multiple imputation. Table 2 presents the point estimate for the propensity score-weighted analysis and each of the sensitivity analysis accompanied by Fig. 3 which includes different types of bootstrap confidence intervals. Supplementary Fig. 6 shows that under the proposed model every prevalence difference from > 0 to 17 percentage point is more compatible with the data than a prevalence difference of 0 and below.

**Table 2:** Estimated prevalence difference in crude, propensity score analysis and sensitivity analysis

	**Prevalence difference; point estimate [95% CI]**^**a**^
Multiply imputed and PS-weighted analysis	8.8 [-1.4; 18.6]^b^
Complete case analysis	
Crude	5.1 [-4.6; 15.0]^b^
PS-weighted	5.1 [-5.0; 16.1]^b^
Sensitivity analyses	
Alternative PS model	8.0 [-1.4; 17.8]
HTQ score threshold for PTSD: 2.3	10.6 [0.6; 18.3]
HTQ score threshold for PTSD: 2.7	7.2 [-3.1; 16.7]
Missing in “SES” in Lebanon are forced to “Don’t know/refuse to answer”	9.5 [-0.1; 19.6]
Missing in “Violence” in Denmark are forced to “Yes”	4.2 [-5.4; 14.8]

Abbreviations: *CI* confidence interval, *HTQ* Harvard Trauma Questionnaire, *PS* propensity score, *SES* socioeconomic status

^a^All numbers are percentage point. The 95-percentile confidence interval is used for all estimates

^b^Based on 999 bootstrap replications, otherwise based on 250 bootstrap replications

^c^Multiply imputed and PS-weighted. Also see Figure 3.

## Discussion

This study aimed at estimating the association between long-distance migration and prevalence of PTSD. The estimate of association corresponded to 87 additional cases of PTSD for every 1000 Syrian refugees that migrated to Denmark instead of Lebanon. The 95-percentile confidence interval indicates that, under the model, the estimate may be as high as 190 additional cases or as low as 4 cases less per 1000 refugees, thus, the estimate did not reach statistical significance, which may be ascribed to the relatively low number of participants.

Our finding is in accordance with a recent study [23] in a non-randomised sample of very heterogeneous groups of Syrian refugees in Lebanon and Norway where “Host country, Norway” was associated with an average increase in HTQ-score of 3% (95% CI [-5%; 12%]) when adjusting for age and sex; however, the study did not provide estimates of how this translated into absolute number of participants with PTSD. With a mean HTQ-score of 1.49 in the study population in Lebanon the number of additional cases in Norway due to “host country” would be marginal. The study did not account for time since arrival in the host country neither by design nor in the statistical analysis. Two studies have been conducted in non-randomised samples of Syrian refugees in Sweden and Turkey [24, 51], both did not take into account time since arrival in the host country. In keeping with our results, Hall and Kahn [51] found that, after adjusting for exposure to trauma, the probability of reporting symptoms of PTSD was higher in the Swedish study population compared with the Turkish study population, however, in opposition to this Chung et al*.* [24] found that the crude prevalence estimate of reporting PTSD symptoms was higher in the Turkish study population compared with the Swedish study population. While dissimilar in aim and methodological approach, the differences in the reported results may also be a result of the non-random sampling design.

The underlying cause for the observed increase in PTSD prevalence in Syrian refugees after long-distance migration has never been investigated. Some studies hypothesise that great distance between populations is associated with difficulty acculturating (e.g. “longing for people, places and things in the homeland” and “not feeling part of one’s surrounding or social structure”) which in turn is associated with worse health outcomes including mental health [22, 52–54], for example, acculturative stress predicted PTSD in a small non-random sample of Bosnian refugees living in Austria for a mean of 18 years [55] and in a large random sample of Syrian refugees living in Sweden for more than 3 years [56]. The present study is a first step in separating out the health effects of the migration from the post-migratory phase in a refugee population: By design, the study populations had stayed in Lebanon or Denmark for a very short time (about 5 months) and the effect of cumulative acculturative stress was minimised but “acute” stressors such as insecurity about the daily living and unfamiliar customs may play a role.

The prevalence estimates of PTSD was high in both Lebanon (55%) and Denmark (60%) compared with previous studies of Syrian refugees [13, 56–59], although higher PTSD prevalence have also been reported [60]. Informally, there does not seem to be a clear relation to host country, study design and utilised instruments for assessing PTSD. In all studies, Syrian refugees had stayed more than a year in the host country at time of inclusion. The randomised design and the very high participation rate in the present study strengthen the confidence in the prevalence estimate.

### Limitations

There are several points of limitations in the current study. Firstly, the targeted sample size was not reached in Lebanon nor in Denmark due to structural and political obstacles as well as the outbreak of a global pandemic during the data collection. Still, a positive association is more compatible with the data than the opposite (see Supplementary Fig. 6). Secondly, the interpretation of the association between long-distance migration and PTSD is limited by the cross-sectional study design. Most importantly, the time-order may be reversed so that instead of long-distance migration affecting the risk of PTSD it may be that PTSD affects the “risk” of undertaking long-distance migration. Specifically, a positive association between long-distance migration and PTSD was found, thus, a reversal of the time order would mean that individuals with PTSD are more likely to undertake long-distance migration, that is, travel to Denmark instead of Lebanon. Although this cannot be rejected, taking into account the pathophysiology of PTSD with impaired executive function including difficulties in concentrating and planning, we find this interpretation less convincing. Thirdly, we used a self-administered score to assess PTSD and did not have the opportunity to confirm its result with a diagnostic interview by a psychiatrist. The HTQ-score is considered valid across multiple settings and diagnostic classification systems and the sensitivity analysis showed little change in the estimate of association including when changing the HTQ-score cut-off. Fourthly, while we have secured strong control of the most important confounders, the obtained sample size did not allow to add all potential confounders in the analysis. The collapsing of two levels of the socioeconomic status variable and the somewhat coarse measure of variables such as trauma exposure (assessed with one item in the questionnaire) may further be a source for residual confounding in both the multiple imputation and in the propensity score weighted analysis. We provide an alternative DAG to illustrate the associations of additional variables (Supplementary Fig. 2). It shows that the minimally sufficient adjustment set may not differ from what was implemented in our analysis. Fifthly, it was not possible to retrieve or create a precise sampling frame in Lebanon and thus, it was based on best information available at the time, knowing that refugees frequently relocate and there is little registration of individuals outside the formalised refugee camps. We still consider the risk of selection bias low given the randomised sampling design and very high participation rates. Sixthly, the proportion of missing data was high in several variables (Fig. 2) which may bias the results. We found that the data was “everywhere missing-at-random”, i.e. for each partly observed variable any possible missingness pattern was independent of the missing values in the variable in question given the covariates and the observed values of the variable [42], and implemented an advanced type of multiple imputation to counter missing data. We discuss this in detail in the Supplementary Text 2. While adherence to the assumptions of multiple imputation cannot be formally proven, in the sensitivity analyses only gross violation of these assumptions changed the magnitude of the estimate while preserving the direction of association. Finally, the proportion that refused to answer, or did not know what to answer, in the question on socioeconomic status was high and differed between the study population in Denmark (22%) and in Lebanon (7%). After discussing this variable with Syrian natives, we speculate that “socioeconomic status” was not defined in sufficient detail for all participants to be able to reflect on the question; however, we do not have an explanation for the difference between Lebanon and Denmark. If participants in Denmark were more likely to misrepresent or hide high socioeconomic status the PTSD prevalence difference after long-distance migration would be even higher.

### Implications for future studies

Future research should concentrate to confirm the association, preferably acquiring a larger sample size. Also of interest is the transportability to other groups of refugees and to investigate the underlying factors contributing to the association, for example examine the different measures of “distance” (cultural, geographical etc.). We note that the direction of the association was consistent across different thresholds for PTSD (effectively lowering and raising the estimated prevalence for PTSD in the study population), different propensity score models and when imposing violation of underlying assumptions of multiple imputation. We hypothesise that the association will be reproducible at least in comparable populations and settings—for example newly arrived adult refugees from a Middle Eastern country to a Western European country.

## Conclusions

In this study, the prevalence of PTSD was high in newly arrived Syrian refugees in both Lebanon and Denmark. In the multiply imputed and propensity score-weighted analysis, the prevalence of PTSD increased with 87 (95-percentile CI [-4; 190]) additional cases of PTSD for every 1000 Syrian refugees that migrated to Denmark instead of Lebanon. Practitioners may take into consideration “long-distance migration” in refugee health screenings and in particular when assessing the risk of PTSD. With the present study we begin to study the association between long-distance migration and mental health and provide the foundation upon which future studies could build.

## Supplementary Information

**Additional file 1: Supplementary Material 1. **STROBE Statement—Checklist. **Supplementary Figure 1.** Directed acyclic graph. **Supplementary Figure 2.** Alternative directed acyclic graph. **Supplementary Figure 3.** Balance plots. **Supplementary Figure 4.** Violin plot of number of children against the participant’s age. **Supplementary Figure 5.** Systolic blood pressure against the participant’s age. **Supplementary Figure 6.** Consonance functions, intervals at every level. **Supplementary Table 1.** The three propensity score models. **Supplementary Table 2.** The predictor matrix for the SMC-FCS multiple imputation. **Supplementary Table 3.** Non-responders: Basic demographics and reason. **Supplementary Text 1.** The background questionnaire. **Supplementary Text 2.** Details about the statistical analysis

## Data Availability

The data that support the findings of this study are available on request from the corresponding author AHE. The data are not publicly available due to them containing information that could compromise research participant privacy and content. The computing code for all parts of the data cleaning, analysis, and plotting is publicly available from the first author’s Github repository: https://github.com/eiset/ARCH. This repository also contains all the exploratory plots produced.

## Abbreviations

HTQ: The Harvard Trauma Questionnaire
PTSD: Post-traumatic stress disorder
SES: Socio-economic status
