# Supplementary material for: The association between long-distance migration and PTSD prevalence in Syrian refugees
Source: BMC Psychiatry. 2022 May 27;22:363. doi: 10.1186/s12888-022-03982-4 (PMC9137139; doi:10.1186/s12888-022-03982-4)
Supplement: Supplementary file 1 — Additional file 1: Supplementary Material 1. STROBE Statement—Checklist. Supplementary Figure 1. Directed acyclic graph. Supplementary Figure 2. Alternative directed acyclic graph. Supplementary Figure 3. Balance plots. Supplementary Figure 4. Violin plot of number of children against the participant’s age. Supplementary Figure 5. Systolic blood pressure against the participant’s age. Supplementary Figure 6. Consonance functions, intervals at every level. Supplementary Table 1. The three propensity score models. Supplementary Table 2. The predictor matrix for the SMC-FCS multiple imputation. Supplementary Table 3. Non-responders: Basic demographics and reason. Supplementary Text 1. The background questionnaire. Supplementary Text 2. Details about the statistical analysis [file 12888_2022_3982_MOESM1_ESM.pdf]

Supplementary material for the research article

# The association between long-distance migration and PTSD prevalence in Syrian refugees

**Table of Contents**

Supplementary Material 1: STROBE Statement—Checklist....2
Supplementary Figure 1: Directed acyclic graph....5
Supplementary Figure 2: Alternative directed acyclic graph....6
Supplementary Text 1: The background questionnaire....7
Supplementary Text 2: Details about the statistical analysis....8
The propensity score-weighted estimate of association....8
The multiple imputation modelling of missing data....8
Supplementary Table 1: The three propensity score models....10
Supplementary Table 2: The predictor matrix for the SMC-FCS multiple imputation....11
Supplementary Figure 3: Balance plots....12
Supplementary Figure 4: Violin plot of number of children against the participant's age....13
Supplementary Figure 5: Systolic blood pressure against the participant's age....14
Supplementary Table 3: Non-responders: Basic demographics and reason....15
Supplementary Figure 6: Consonance functions, intervals at every level....16

## Supplementary Material 1: STROBE Statement—Checklist

of items that should be included in reports of cross-sectional studies

| | Item No | Recommendation | Page No |
|---|---|---|---|
| **Title and abstract** | 1 | (*a*) Indicate the study's design with a commonly used term in the title or the abstract | 1 |
| | | (*b*) Provide in the abstract an informative and balanced summary of what was done and what was found | 2 |
| **Introduction** | | | |
| Background/rationale | 2 | Explain the scientific background and rationale for the investigation being reported | 4 |
| Objectives | 3 | State specific objectives, including any prespecified hypotheses | 4 |
| **Methods** | | | |
| Study design | 4 | Present key elements of study design early in the paper | 4 |
| Setting | 5 | Describe the setting, locations, and relevant dates, including periods of recruitment, exposure, follow-up, and data collection | 4-5 |
| Participants | 6 | (*a*) Give the eligibility criteria, and the sources and methods of selection of participants | 4 |
| Variables | 7 | Clearly define all outcomes, exposures, predictors, potential confounders, and effect modifiers. Give diagnostic criteria, if applicable | 5-6 & Supplement |
| Data sources/ measurement | 8* | For each variable of interest, give sources of data and details of methods of assessment (measurement). Describe comparability of assessment methods if there is more than one group | 5-6 & Supplement |
| Bias | 9 | Describe any efforts to address potential sources of bias | 7-8 & Supplement |
| Study size | 10 | Explain how the study size was arrived at | 6-7 & Supplement |
| Quantitative variables | 11 | Explain how quantitative variables were handled in the analyses. If applicable, describe which groupings were chosen and why | 7-8 & Supplement |
| Statistical methods | 12 | (*a*) Describe all statistical methods, including those used to control for confounding | 7-8 & Supplement |
| | | (*b*) Describe any methods used to examine subgroups and interactions | 7-8 & Supplement |
| | | (*c*) Explain how missing data were addressed | 7-8 & |

| | | | |
|---|---|---|---|
| | | | Supplement |
| | | (*d*) If applicable, describe analytical methods taking account of sampling strategy | NA |
| | | (*e*) Describe any sensitivity analyses | 7-8 & Supplement |
| **Results** | | | |
| Participants | 13* | (a) Report numbers of individuals at each stage of study—eg numbers potentially eligible, examined for eligibility, confirmed eligible, included in the study, completing follow-up, and analysed | 8 & fig. 1 |
| | | (b) Give reasons for non-participation at each stage | Fig. 1 & Suppl. Table 3 |
| | | (c) Consider use of a flow diagram | Fig. 1 |
| Descriptive data | 14* | (a) Give characteristics of study participants (eg demographic, clinical, social) and information on exposures and potential confounders | 8 & table 1 |
| | | (b) Indicate number of participants with missing data for each variable of interest | Table 1 & Fig. 2 |
| Outcome data | 15* | Report numbers of outcome events or summary measures | 9 & Table 1 |
| Main results | 16 | (*a*) Give unadjusted estimates and, if applicable, confounder-adjusted estimates and their precision (eg, 95% confidence interval). Make clear which confounders were adjusted for and why they were included | 9, Table 2 and Figure 3 |
| | | (*b*) Report category boundaries when continuous variables were categorized | Table 1 |
| | | (*c*) If relevant, consider translating estimates of relative risk into absolute risk for a meaningful time period | NA |
| Other analyses | 17 | Report other analyses done—eg analyses of subgroups and interactions, and sensitivity analyses | 9 |
| **Discussion** | | | |
| Key results | 18 | Summarise key results with reference to study objectives | 9 |
| Limitations | 19 | Discuss limitations of the study, taking into account sources of potential bias or imprecision. Discuss both direction and magnitude of any potential bias | 11-12 |
| Interpretation | 20 | Give a cautious overall interpretation of results considering objectives, limitations, multiplicity of analyses, results from similar studies, and other relevant evidence | 9-11 |
| Generalisability | 21 | Discuss the generalisability (external validity) of the study results | 12-13 |
| **Other information** | | | |
| Funding | 22 | Give the source of funding and the role of the funders for the present | 14 |

| | | | |
|---|---|---|---|
| | | study and, if applicable, for the original study on which the present article is based | |

*Give information separately for exposed and unexposed groups.

Note: An Explanation and Elaboration article discusses each checklist item and gives methodological background and published examples of transparent reporting. The STROBE checklist is best used in conjunction with this article (freely available on the Web sites of PLoS Medicine at http://www.plosmedicine.org/, Annals of Internal Medicine at http://www.annals.org/, and Epidemiology at http://www.epidem.com/). Information on the STROBE Initiative is available at www.strobe-statement.org.

## Supplementary Figure 1: Directed acyclic graph

Assumptions about the association between long-distance-migration and post-traumatic stress disorder

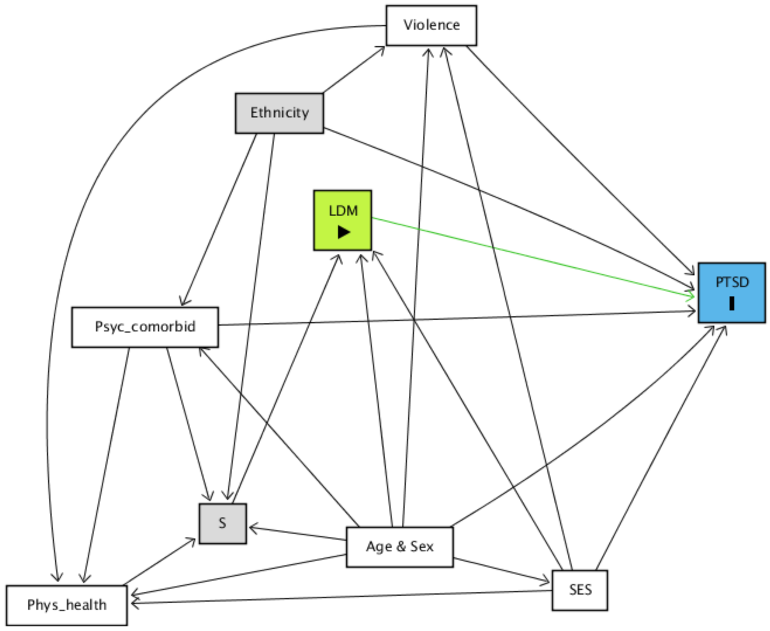


*Abbreviations: S, selection variable, variables going into this define the study population; LDM, long-distance migration (the exposure of interest); PTSD, post-traumatic stress disorder (the outcome of interest); SES, socio-economic status; Psyc_comorbid, psychiatric co-morbidity; Phys_health, physical health; Violence, exposure (directly or indirectly) to violence. Ethnicity is used to capture genetics background, physical phenotype, parental physical phenotype and cultural context. Likewise, age and sex is collapsed in this depiction after securing no loss of information. Colour code: Green, exposure; Blue, outcome; Grey, covariates that are controlled for by design; White, other covariates.*

## Supplementary Figure 2: Alternative directed acyclic graph

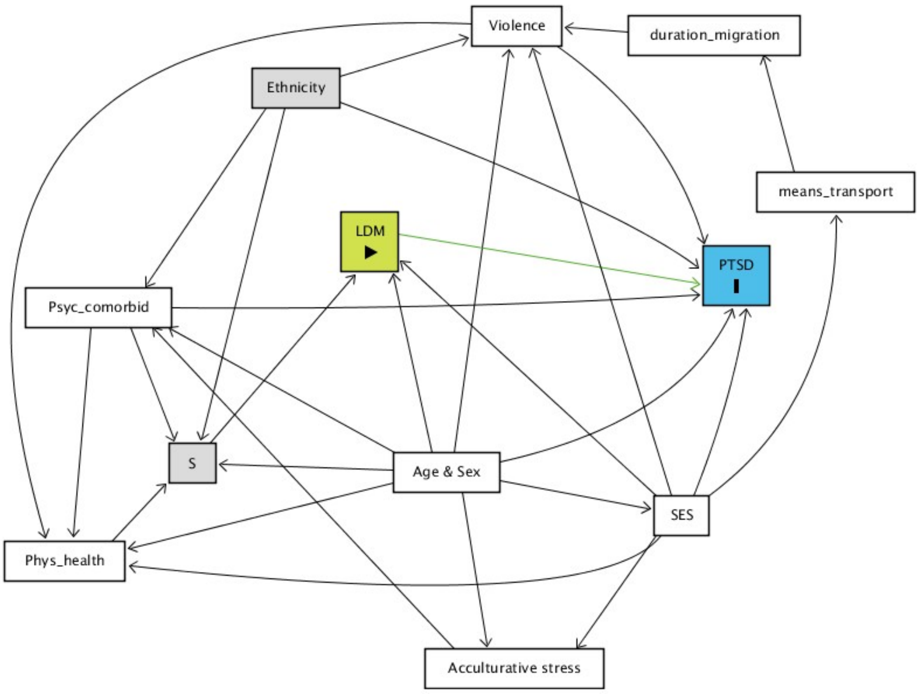


*In comparison ot Supplementary Figure 1 there are added variables on duration of migration, means of transportation and acculturative stress. The minimally sufficient adjustment set is unchanged.*

## Supplementary Text 1: The background questionnaire

Below, we reproduce parts of the questionnaire in English translated by the authors of the manuscript. This translation has never been used for data collection and has not been validated by a translator.

1. About you and your family

1.a Your sex (circle correct answer): male female

1.b Your age (in years):

1.c Your marital status (circle correct answer): unmarried married widowed other

1.d How many children do you have (circle correct answer): 0 1 2 3 4 5or more

2. About your education

2.a What is your highest obtained educational level (circle correct answer): none elementary gymnasium higher

2.b How many years of education have you had (in years):

2c How would you rank your social status before leaving Syria (circle correct answer): below average on average above average do not know

3. About your health

3.a Do you smoke tobacco (circle correct answer): yes no, but previously no, never

4. About your migration

4.a On what date did you leave Syria (year, month, day, as precisely as you remember):

4.b On what date did you arrive in [Lebanon/Denmark] (year, month, day, as precisely as you remember):

4.c What means of travel did you use to get from Syria to [Lebanon/Denmark] (circle all applicable): airplane car bus train boat walked long distances other

4.d During your travel, did you experience violence or abuse (circle all applicable): yes, first hand yes, second hand no

## Supplementary Text 2: Details about the statistical analysis

### The propensity score-weighted estimate of association

Variables to include to control confounding and potential confounding were age, sex, mental health co-morbidity, experience of violence and SES. Three propensity score models with increasing complexity was proposed (Supplementary Table 1) and covariate balance in the exposure groups was assessed with no truncation, truncation at 1st and 99th percentile and at 5th and 95th percentile (Supplementary Figure 2). The simplest model with the least amount of truncation that obtained acceptable balance, defined as an absolute mean difference less than or equal to 0.10 for all covariate, was used for the propensity score analysis. From Supplementary Figure 2 it is evident that the simple model with truncation at 1st and 99th percentile obtained balance on all covariates. For example, for the sex variable, in Lebanon approximately 73% of participants were female, whereas this was the case for 47% of the participants included in Denmark, resulting in a difference of 26 percentage point. In the re-weighted "pseudo-population" the mean difference was reduced to 4 percentage point.

### The multiple imputation modelling of missing data

The substantive model compatible fully conditional specification (SMC-FCS) implementation of multiple imputation was utilized. Here, the substantive model (here the propensity score model) entered separately and a "prediction model" (used here to describe a model to predict the value of the covariate in question given the other covariates and possible auxiliary variables) was specified for each partially observed covariate and any auxiliary variables. The extensive exploratory analysis including plots and tables are available from https://github.com/eiset/ARCH and the resulting "response-and-predictor matrix" and details of how each variable was treated is supplied in "The predictor matrix" below.

An example of the discussion of missing data follows using the variables "Children" (i.e. the participant's number of offspring) and "Age" (i.e. the participant's age in years at time of inclusion). While Children is not in the substantive model (i.e. does not enter the propensity score model) and might be considered less important in this analysis, it holds valuable information on a very important variable: Age (Supplementary Figure 3). Therefore, it enters as an auxiliary variable in the prediction model for Age. Before doing so, the missing data in Children must be imputed by specifying a prediction model with Children as the response (Supplementary Table 2) and the validity of multiple imputation must be discussed for Children. In our analysis, the assumption of ignorable missingness mechanism requires that the missingness mechanism is at least "everywhere missing-at-random", i.e. that any possible missingness pattern of Children is independent of the missing values in Children given the covariates and the observed values for Children. If it is more likely that an individual with no children did not answer the question and if no other variables are considered, then the missingness mechanism is at best "realized missing-at-random" (if all instances of Children = 0 are realized, which we cannot know). In the case of Children, among others low age seems to increase the probability of data being missing (Supplementary Figure 3). See Supplementary Table 2 above for the other variables that were included as predictors in modeling Children based on subject matter knowledge and the exploratory analysis https://github.com/eiset/ARCH. Assuming independent and identical missingness mechanism, we considered the possible missingness patterns for one individual, and judged that the "everywhere missing-at-random" assumption was well approximated. Next, the assumption of correctly specified multiple imputation depends on whether the substantive

model of interest is correctly specified and whether it is possible to build a multiple imputation model that captures the entirety of the data generating process. Again, this relies on subject matter insight, a thorough discussion of the proposed model and a deep understanding of the applied methods.

For the imputation of the partly observed variable Age, which is in the substantive model, we again rely on sufficient information being observed so that we may predict the missingness pattern and assume that the missingness mechanism is approximately "everywhere missing-at-random". A strong predictors of age is systolic blood pressure (Supplementary Figure 4). Systolic blood pressure is not in our propensity score model, thus, it is an auxiliary variable. It has missing values (Figure 1 in the main text) and so also needs imputing…

## Supplementary Table 1: The three propensity score models

Covariates in each of the three propensity score models and how they enter. “Long-distance migration” is the response in all models

| | **Enter as** | **Model 1: “simple”** | **Model 2: “intermediate”** | **Model 3: “complex”** |
|---|---|---|---|---|
| Age | 3-knots restricted cubic spline | x | x | x |
| Sex | Dichotomous | x | x | x |
| WHO-5 | 3-knots restricted cubic spline | x | x | x |
| Violence | Dichotomous | x | x | x |
| SES | 3-level ordinal | x | x | x |
| Sex & age | Interaction, no transformation | | x | x |
| Sex & WHO-5 | Interaction, no transformation | | | x |
| Age & WHO-5 | Interaction, no transformation | | | x |

*Abbreviations: Interact., interaction term; WHO-5, the WHO-5 quality of life scale; SES, socio-economic status; Violence, experience (direct or indirect) of violence during migration.*

## Supplementary Table 2: The predictor matrix for the SMC-FCS multiple imputation

| | | **Predictors** | | | | | | | | | | | | | | | | | | | | | | | | |
|---|---|---|---|---|---|---|---|---|---|---|---|---|---|---|---|---|---|---|---|---|---|---|---|---|---|---|
| **Type** | **Response** | migr | age_log | age | age_sb | sex | sexFemale:age | who_sqrt | who | who_sb | who:sexFemale | who:age | viol | ses | ptsd | ptsd_sb | edu | child | smok | bp_log | bp | bp_sb | hgt_log | hgt | hgt_sb | mari |
| b | ***migr*** | | | | | | | | | | | | | | | | | | | | | | | | | |
| c | ***age_log*** | | | | | | | | | | | | | 1 | 1 | 1 | 1 | 1 | | | 1 | 1 | | | | 1 |
| pas | age | | 1 | | | | | | | | | | | | | | | | | | | | | | | |
| pas | age_sb | | | 1 | | | | | | | | | | | | | | | | | | | | | | |
| b | ***sex*** | | | | | | | | | | | | | | 1 | 1 | | | 1 | | 1 | 1 | | 1 | 1 | 1 |
| pas | **sexFemale:age** | | | 1 | | 1 | | | | | | | | | | | | | | | | | | | | |
| c | ***who_sqrt*** | | | 1 | 1 | 1 | 1 | | | | | | 1 | 1 | 1 | 1 | | | | | | | | | | |
| pas | who | | | | | | | 1 | | | | | | | | | | | | | | | | | | |
| pas | who_sb | | | | | | | | 1 | | | | | | | | | | | | | | | | | |
| pas | **who:sexFemale** | | | | | 1 | | | 1 | | | | | | | | | | | | | | | | | |
| pas | **who:age** | | | 1 | | | | | 1 | | | | | | | | | | | | | | | | | |
| b | ***viol*** | | | 1 | 1 | 1 | 1 | | 1 | 1 | 1 | | | | 1 | 1 | | | | | | | | | | |
| o | ***ses*** | | | 1 | 1 | 1 | 1 | | 1 | 1 | 1 | | | | 1 | 1 | | 1 | | | | | | | | 1 |
| c | ***ptsd*** | | | 1 | 1 | 1 | 1 | | 1 | 1 | 1 | 1 | 1 | 1 | | | | | | | | | | | | |
| pas | ptsd_sb | | | | | | | | | | | | | | 1 | | | | | | | | | | | |
| o | *edu* | | | 1 | 1 | 1 | 1 | | | | | | | 1 | | | | | | | | | | | | 1 |
| o | *child* | 1 | | 1 | 1 | 1 | 1 | | | | | | | 1 | | | 1 | | | | 1 | 1 | | | | 1 |
| b | *smok* | 1 | | | | 1 | | | | 1 | 1 | | 1 | | | | | | | | 1 | 1 | | 1 | 1 | |
| c | *bp_log* | | | 1 | 1 | 1 | 1 | | | | | | | | | | | | | | | | | | | |
| pas | bp | | | | | | | | | | | | | | | | | | | 1 | | | | | | |
| pas | bp_sb | | | | | | | | | | | | | | | | | | | | 1 | | | | | |
| c | *hgt_log* | | | | | 1 | | | | | | | | | 1 | 1 | | | 1 | | 1 | 1 | | | | |
| pas | hgt | | | | | | | | | | | | | | | | | | | | | | 1 | | | |
| pas | hgt_sb | | | | | | | | | | | | | | | | | | | | | | | 1 | | |
| u | *mari* | | | | | 1 | | | | | | | | | | | | 1 | | | | | | | | |

*The predictor matrix details how each partially observed variable was imputed. Horizontally the variables enter as the response, vertically the variables enter as the predictor when the corresponding cell is "1". The "type" column indicate how the variables should be modelled when entering as the response (b, binary; c, continuous; pas, passive; o, ordered/ordinal; u, unordered/nominal). Variables ending on "_sb" are modelled as restricted cubic spline (spline basis); ":" indicates interaction between the variables on each side. Variables in bold are part of the substantive model (the propensity score model); variables in italic are actively imputed.*

## Supplementary Figure 3: Balance plots

For each of the combinations of the three propensity score model and the three cut-offs for weight truncation

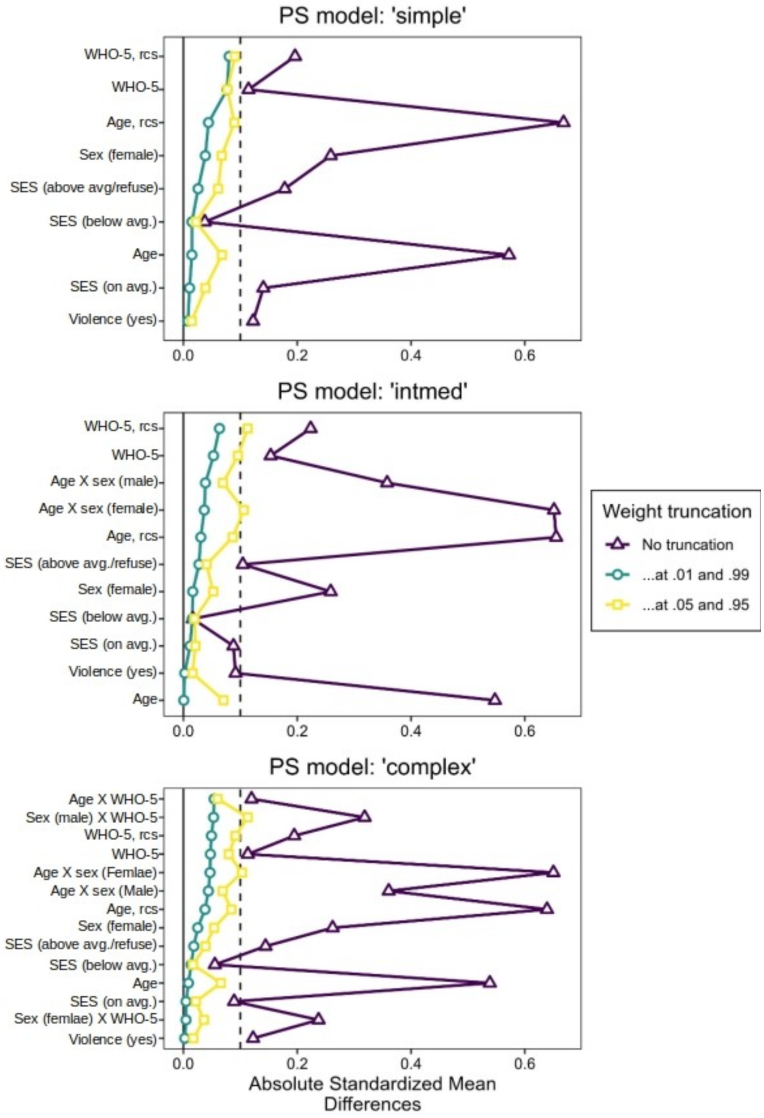


*Abbreviations: WHO5, WHO (five) Well-being Index; rcs, three-knots restricted cubic spline term; SES, socioeconomic status; avg, average; Violence, exposure (directly or indirectly) to violence; X, interaction term.*

**Supplementary Figure 4: Violin plot of number of children against the participant's age**

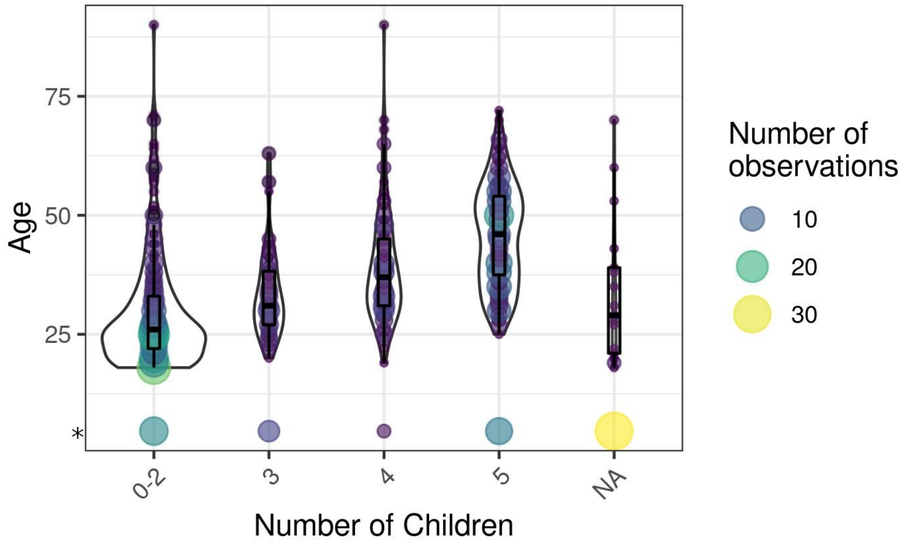


**Missing values in Age are plotted as (minimum value minus mean absolute deviation). The relative point size and colour gradient indicate the density of observations at each space. It is clear that the number of children is a fairly good predictor of age (e.g. in the case of 2 versus 3 children the "best guess" of the age of a participant with missing information on age would change from approximately 25 years old to approximately 33 years old).*

**Supplementary Figure 5: Systolic blood pressure against the participant's age**

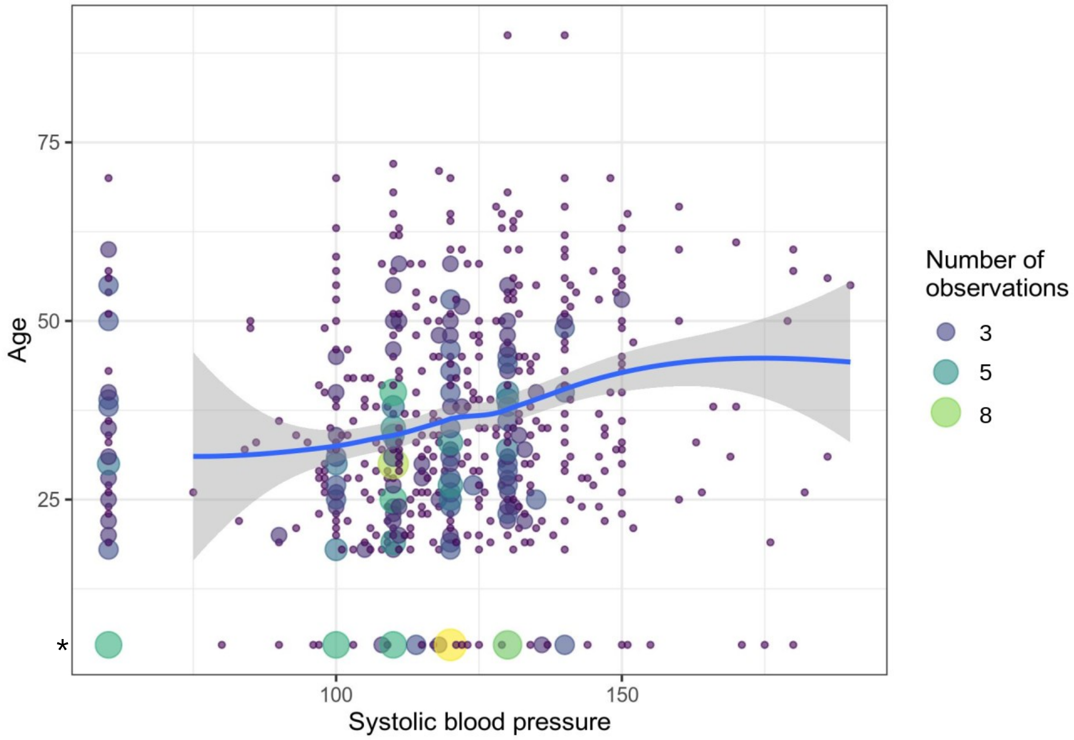


**Missing values are plotted as (minimum value minus mean absolute deviation). The relative point size and colour gradient indicate the density of observations at each space. It is clear that the blood pressure is a strong predictor of age (in accordance with subject matter knowledge).*

## Supplementary Table 3: Non-responders: Basic demographics and reason

| Number of individuals | | Denmark | Lebanon |
|---|---|---|---|
| Refused to participate | | 8 | 31 |
| | Sex, female | 2 | 5[#] |
| | Age[†] | All between 20 and 30 years old | All younger than 50 years old |
| Reasons[‡] | | | |
| | Mistrust | 5 | 1 |
| | No time | 2 | 4 |
| | No personal gain | 0 | 2 |
| | No answer | 1 | 24 |

*[#]Sex was only recorded for eight non-participants in Lebanon. [†]Only few non-participants provided age (two in Denmark and two in Lebanon), thus a best guess is provided. [‡]Reasons provided from the non-participants, aggregated into smallest possible number of strata.*

## Supplementary Figure 6: Consonance functions, intervals at every level

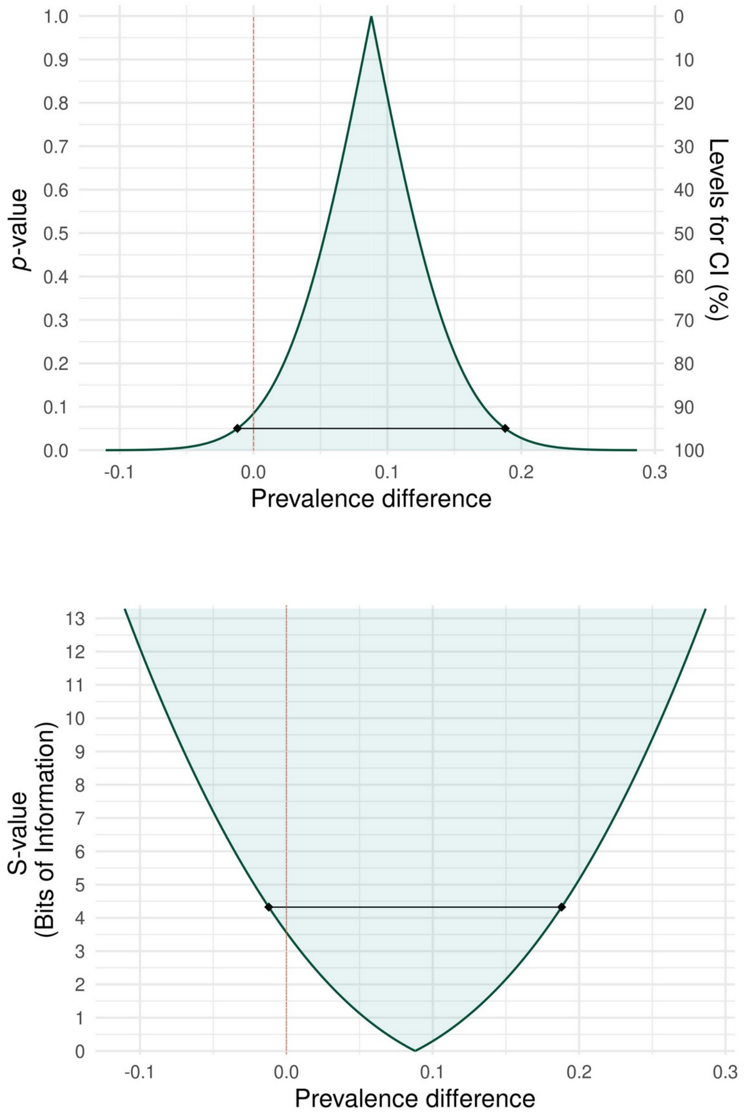


*Top panel: Plotting p-values and corresponding CI against possible values of prevalence difference. Bottom panel: S-values against possible values of prevalence difference. The horizontal black line indicates the 95% confidence interval. The vertical red line indicates the "null-value", i.e. no prevalence difference between those that undertook long-distance migration to Denmark and those who migrated to Lebanon. Under the background assumptions every prevalence difference from >0 to 17 percentage point has less information against it than a value of 0 and below.*
